# Cross-sectional study on the health of workers exposed to occupational noise in China

**DOI:** 10.1371/journal.pone.0305576

**Published:** 2024-06-25

**Authors:** Nan Jin, Wei He, Huadong Zhang, Huaxin Deng, Qi Zhao, Fengqiong Chen, Xiaoni Zhong, Fang Yuan

**Affiliations:** 1 Department of Occupational Health and Radiation Health, Chongqing Center for Disease Control and Prevention, Chongqing, China; 2 Chongqing Jiulongpo District Center for Disease Control and Prevention, Chongqing, China; 3 School of Public Health, Chongqing Medical University, Chongqing, China; Drexel University School of Public Health, UNITED STATES

## Abstract

**Objective:**

This study aimed to understand the health of workers exposed to occupational noise and explore the influencing factors related to workers’ health, especially the impact of noise on workers’ hearing. This work can provide a basis for formulating relevant measures for occupational noise prevention and control in the future.

**Methods:**

On the basis of the key occupational disease monitoring project in Chongqing, China, in 2021, the data of 1125 workers exposed to occupational noise were analyzed. Data included demographic information, occupational history, clinical physical examination information, and noise detection information of the working environment. Chi-square test and multifactorial logistic regression were used for statistical analysis.

**Results:**

The prevalence rates of abnormal electrocardiogram (ECG), blood pressure (BP), and pure tone audiometry (PTA) were 21.9% (246/1125), 27.8% (313/1125), and 18.0% (202/1125), respectively. Male workers accounted for 78.8%. Compared with male workers, female workers had a lower prevalence of abnormal PTA (OR = 0.28, 95% CI = 0.16–0.50). Workers working in medium enterprises had a lower prevalence of abnormal BP than workers in micro enterprises (OR = 0.36, 95% CI = 0.19–0.66). The prevalence of abnormal BP and PTA of workers increased with age. After adjusting for age, sex, and body mass index, the prevalence of abnormal ECG of mining workers was higher than that of manufacturing workers (OR = 1.54, 95% CI = 1.07–2.24), and the prevalence of abnormal PTA had a rising trend with the increase in noise exposure value.

**Conclusion:**

Noise-exposed workers have a high prevalence of abnormal ECG, BP, and PTA, and factors such as age, enterprise size, and workplace noise exposure are correlated with the aberrant health of workers. Governments, enterprises, and individuals need to attach great importance to the possible adverse effects of noise. They must also actively adopt various effective measures to protect the occupational safety and health of workers.

## 1. Introduction

With the rapid development of global industrialization, various health problems caused by occupational noise exposure have become an increasingly serious public health concern [1]. As an environmental stress source, occupational noise has an impact on various organs and systems of workers, such as the digestive system, respiratory system and immune system [1, 2]. Among the numerous impacts, occupational noise has a particularly prominent influence on workers’ hearing and cardiovascular system [3]. Occupational noise mainly causes hearing loss of workers, especially high-frequency hearing, and it is attributed to cardiovascular diseases such as hypertension and myocardial infarction of workers [3–8].

Prolonged exposure to noise can lead to excessive stimulation of inner ear hair cells, resulting in structural damage and the consequent attenuation and distortion of incoming auditory stimuli, which is one of the main reasons for hearing loss [9, 10]. Noise is also related to cardiovascular disease, and the association can be explained by the biochemical changes related to the mechanisms of stress, in brief, an increase in the level of hormones such as cortisol, adrenaline, and noradrenaline in response to the stress caused by noise can result in peripheral vasoconstriction, increased heart rate, and increased arterial blood pressure (BP) [11]. BP and electrocardiogram (ECG) measurements have become the most common research indicators in the cardiovascular system [7]. In addition, a large number of research results have demonstrated that factors such as age, sex, and job type may be related to workers’ hearing loss and cardiovascular disease [12–15].

Exposure to harmful noise is one of the most common occupational risks, with approximately 600 million workers worldwide exposed to noise [16, 17]. About 25% of the working population in Tunisia are exposed to high levels of noise over 85 decibels (dB) [6]. Nearly 22 million workers in the United States are also exposed to high noise levels. The study found that about 33% of adults with a history of occupational noise exposure in the United States can provide audiological evidence of corresponding hearing loss, and about one-quarter of workers have been exposed to occupational noise in their career [18, 19]. Noise-induced hearing loss (NIHL) caused by occupational noise, especially occupational noise-induced deafness, has become one of the most common occupational diseases in most industrialized countries [20]. Meta-analyses have been conducted to quantitatively assess the exposure–response link for ambient noise and health effects (hypertension and ischemic heart diseases, including myocardial infarction). The investigators reported increases in risk of between 7% and 17% per 10 dB increase in equivalent noise level [15, 21].

At present, occupational noise-induced deafness has become the second largest occupational disease in China after occupational pneumoconiosis. According to China’s annual physical examination of noise-exposed workers, about 50% of workers with more than 5 years of service in noise operations, especially workers exposed to noise above 85 dB, have varying degrees of hearing loss, and the proportion of noise-induced deafness among workers with more than 20 years of working experience is about 1%–5% [22]. A meta-analysis from China showed that workers exposed to noise have a 2.55 times higher risk of developing hypertension and 2.27 times higher risk of abnormal ECG compared with the control group [7].

The Chinese government attaches great importance to noise prevention and control. To protect the hearing health of residents, especially workers, China has enacted the corresponding law on the prevention and treatment of occupational diseases and implemented the Law of the People’s Republic of China on Prevention and Control of Pollution From Environmental Noise on June 5, 2022 [23].

Long-term exposure to noise above acceptable thresholds reduces worker concentration, performance, awareness, and efficiency, and it can even lead to accidents [16]. Thus, enterprises and society need to bear not only the capacity reduction and cost increase but also the heavy disease burden caused by occupational noise exposure [24, 25].

China is a developing country with a large number of workers. Health problems such as hearing loss caused by occupational noise have brought a huge burden of disease to China. This study aimed to investigate the health of noise-exposed workers and explore the influencing factors related to workers’ health, especially the impact of noise on workers’ hearing, to provide a basis for formulating noise prevention and control measures in the future.

## 2. Materials and methods

### 2.1. Study subjects

The survey population of this study was obtained from the Chongqing Municipal Key Occupational Disease Surveillance Project in 2021, which included 5483 active monitoring data, where 1149 matched with workplace noise monitoring data. Severely missing and incorrect data were excluded, and 1125 data were included in the analysis, of which 74 data lacked specific noise values (**S1 Fig**). We began preliminary collation and analysis of the data in November 2022. The studies involving human participants were reviewed and approved by the Medical Ethics Committee of Chongqing Center for Disease Control and Prevention (May 17, 2019) and the Academic Management Committee of Chongqing Center for Disease Control and Prevention (May 17, 2019). Patient consent was waived due to the retrospective nature of their data.

### 2.2. Data collection

The occupational health examination program was conducted in accordance with the Chinese Technical Specifications for Occupational Health Surveillance (GBZ 188–2014) [26]. Every year, the government will select some enterprises within the region based on industry types to arrange for employees to undergo health examinations at specific medical institutions. The data collected included the demographic information, occupational history, clinical physical examination information, and detection results of noise in the working environment. The occupational history included the category and size of the work enterprise, position, contact start time, and contact end time. The noise value of the individual working environment was obtained by matching the information of the enterprise, position, and type of work with the monitoring results of the working environment noise.

Workplace noise was measured in accordance with the Chinese Occupational Health Standards (GBZ/T 189.8–2007) by using a sound-level meter to collect workplace noise at designated measurement points in accordance with relevant operational requirements [27]. The classification of industrial categories refers to National Economic Industry Classification (GB/T 4754–2017) [28]. The enterprise size is mainly divided according to the type of enterprises, operating income, and number of employees of the enterprise; the reference document is Statistical Classification of Large, Medium, Small and Micro Enterprises (2017) [29].

The institutions responsible for workers’ occupational health examination and workplace noise monitoring were all public institutions with corresponding testing qualifications, such as occupational health examination hospitals and occupational disease prevention and control centers.

Body mass index (BMI) was defined as weight/height^2^: normal (≤24.9 kg/m^2^), overweight (25.0–29.9 kg/m^2^), and obese (≥30.0 kg/m^2^). The content of clinical physical examination mainly included ECG examination, BP measurement, and pure tone audiometry (PTA) examination. ECG was performed by using a conventional 12-lead ECG and interpreted in accordance with the international ECG standards. All results were defined as abnormal, except for "normal electrocardiogram" and "sinus rhythm". BP was mainly measured with an electronic sphygmomanometer (Yuwell, China). In accordance with the standard classification of the World Health Organization, workers with systolic BP >140 mmHg or diastolic BP >90 mmHg were defined as hypertensive patients. PTA was performed in accordance with the national standard of the People’s Republic of China “Pure-tone air and bone conduction audiometry” (GB/T 16296.1–2018) [30]. The subjects’ hearing was measured with a pure tone audiometer in a room with a background noise level <25 dB, and the two ears of the subject were tested with rising pure tones at frequencies of 0.5, 1, 2, 3, 4, and 6 kHz. The lowest signal strength determined was used as the final threshold for each ear, and an average threshold of 3, 4, and 6 kHz was used to determine the high-frequency hearing threshold status. Binaural high-frequency average hearing threshold ≤40 dB was defined as normal [12, 26].

The workplace noise monitoring program and noise grading were conducted in accordance with the China Workplace Occupational Hazards Operational Grading (GBZ/T 229.4–2012) [31], which measures normalization of equivalent continuous A-weighted sound pressure level to a normal 8 h working day (*L*_EX,8h_) or normalization of equivalent continuous A-weighted sound pressure level to a normal 40 h working week (*L*_EX,W_) by using a sound-level meter. Environmental noise value *L*_EX,8h_/*L*_EX,W_ ≥80 dB was defined as exceeding the standard for noise exposure [31].

### 2.3. Statistical analysis

Categorical data were described by frequency and percentage. Data were analyzed by Chi-square test or Fisher’s exact probability method. *p*<0.05 indicated that the difference was statistically significant. Multivariate stepwise logistic regression analysis was used to explore the risk factors for abnormal BP and ECG and increased binaural high-frequency average hearing threshold in noise-exposed workers. Odds ratio (OR) and confidence interval (CI) were used to report the data. In multivariate analysis, the results of ECG, BP, and PTA were used as dependent variables (0 = normal and 1 = abnormal). The variables included in model 1 were age, sex, BMI, exposure time, noise exposure, enterprise size, and industry category. Model 2 adjusted for age, sex, and BMI on the basis of model 1 and changed the noise exposure to noise exposure value grouping, focusing on the study of occupational factors, especially the relationship between the noise and workers’ health. The variables included in model 2 were exposure time, enterprise size, industry category, noise value grouping, with age, sex, and BMI as covariates. SAS 9.4 software (SAS Institute, Cary, NC, USA) was used for statistical analysis.

## 3. Results

### 3.1. Demographic characteristics

Of the 1125 workers included in the final analysis, 78.8% (886/1125) were males, 72.4% (815/1125) were from manufacturing, 74.4% (837/1125) were from small enterprises, and 30.7% (345/1125) workers were overweight **(Table 1)**. The specific noise exposure with different characteristics could be found in **S1 Table**.

**Table 1:** Analysis of occupational health examination results.

Characteristics	Total	ECG				BP				PTA			
		Normal	Abnormal	Chi-Square	*p*-Value	Normal	Abnormal	Chi-Square	*p*-Value	Normal	Abnormal	Chi-Square	*p*-Value
Industry category													
Mining	286 (25.4%)	221 (77.3%)	65 (22.7%)	0.514	0.8	207 (72.4%)	79 (27.6%)	0.028	1.0	218 (76.2%)	68 (23.8%)	13.875	**<0.01**
Manufacturing	815 (72.4%)	638(78.3%)	177 (21.7%)			588 (72.1%)	227 (27.9%)			689 (84.5%)	126 (15.5%)		
Others	24 (2.1%)	20(83.3%)	4 (16.7%)			17 (70.8%)	7 (29.2%)			16 (66.7%)	8 (33.3%)		
Enterprise size													
Micro	118 (10.5%)	89 (75.4%)	29 (24.6%)	0.583	0.7	77 (65.3%)	41 (34.7%)	25.030	**<0.01**	100 (84.7%)	18 (15.3%)	18.110	**<0.01**
Small	837 (74.4%)	656 (78.4%)	181 (21.6%)			586 (70.0%)	251 (30.0%)			665 (79.4%)	172 (20.6%)		
Medium	170 (15.1%)	134 (78.8%)	36 (21.2%)			149 (87.7%)	21 (12.3%)			158 (92.9%)	12 (7.1%)		
Sex													
Male	886 (78.8%)	692 (78.1%)	194 (21.9%)	0.002	1.0	630 (71.1%)	256 (28.9%)	2.385	0.1	699 (78.9%)	187 (21.1%)	28.100	**<0.01**
Female	239(21.2%)	187 (78.2%)	52 (21.8%)			182 (76.2%)	57 (23.8%)			224 (93.7%)	15 (6.3%)		
Age													
<40	257 (22.8%)	194 (75.5%)	63 (24.5%)	1.861	0.4	223 (86.8%)	34 (13.2%)	46.694	**<0.01**	242 (94.2%)	15 (5.8%)	39.000	**<0.01**
40–49.9	383(34.0%)	298 (77.8%)	85 (22.2%)			282 (73.6%)	101 (26.4%)			314 (82.0%)	69 (18.0%)		
≥50	485 (43.1%)	387 (79.8%)	98 (20.2%)			307 (63.3%)	178 (36.7%)			367 (75.7%)	118 (24.3%)		
Noise exposure													
Normal	452 (40.2%)	337 (74.6%)	115 (25.4%)	5.655	**0.02**	332 (73.5%)	120 (26.5%)	0.610	0.4	391 (86.5%)	61 (13.5%)	10.202	**0.01**
Excessive	673 (59.8%)	542 (80.5%)	131 (19.5%)			480 (71.3%)	193 (28.7%)			532 (79.0%)	141 (21.0%)		
Noise value grouping*													
<80	264 (25.1%)	172 (65.2%)	92 (34.8%)	35.062	**<0.01**	188 (71.2%)	76 (28.8%)	3.293	0.3	239 (90.5%)	25 (9.5%)	21.996	**<0.01**
80–84.9	346 (32.9%)	295 (85.3%)	51 (14.7%)			250 (72.3%)	96 (27.7%)			279 (80.6%)	67 (19.4%)		
85–89.9	265 (25.2%)	204 (77.0%)	61 (23.0%)			181 (68.3%)	84 (31.7%)			201 (75.8%)	64 (24.2%)		
≥90	176 (16.8%)	140 (79.5%)	36 (20.5%)			134 (76.1%)	42 (23.9%)			136 (77.3%)	40 (22.7%)		
Exposure time (years)													
≤5	556 (49.4%)	432 (77.7%)	124 (22.3%)	0.272	0.9	405 (72.8%)	151 (27.2%)	1.101	0.6	475 (85.4%)	81 (14.6%)	8.677	**0.01**
5.1–10	327 (29.1%)	255 (78.0%)	72 (22.0%)			229 (70.0%)	98 (30.0%)			259 (79.2%)	68 (20.8%)		
>10	242 (21.5%)	192 (79.3%)	50 (20.7%)			178 (73.6%)	64 (26.4%)			189 (78.1%)	53 (21.9%)		
BMI (kg/m^2^)													
≤24.9	740 (65.8%)	578(78.1%)	162 (21.9%)	0.488	0.8	569 (76.9%)	171 (23.1%)	25.394	**<0.01**	601 (81.2%)	139 (18.8%)	1.408	0.5
25–29.9	345 (30.7%)	268(77.7%)	77 (22.3%)			221 (64.1%)	124 (35.9%)			290 (84.1%)	55 (15.9%)		
≥30	40(3.6%)	33(82.5%)	7 (17.5%)			22 (55.0%)	18 (45.0%)			32 (80.0%)	8 (20.0%)		

ECG: Electrocardiogram examination; BP: Blood pressure examination; PTA: Pure tone audiometry examination

*74 data lacked specific noise values.

### 3.2. Clinical examination results

The prevalence rates of abnormal ECG, BP, and PTA were 21.9% (246/1125), 27.8% (313/1125), and 18.0% (202/1125), respectively. Older workers had a higher prevalence of abnormal BP and PTA. The prevalence rates of abnormal BP and PTA of workers in medium-sized enterprises were lower than those of workers in micro- and small-sized enterprises. The prevalence of abnormal PTA in workers exposed to excessive occupational noise was 21.0% which was higher than 13.5% in their counterparts (**Table 1**). We also explored the correlation among ECG, BP, and PTA, but the results showed no correlation among these three variables.

### 3.3. Multivariate logistic analysis

Model 1 showed that the prevalence of abnormal BP was lower for workers in medium-sized enterprises than for those in micro-sized enterprises (OR = 0.36, 95% CI = 0.19–0.66). Moreover, the prevalence of abnormal BP was higher in workers who were overweight (OR = 1.99, 95% CI = 1.49–2.66) or obese (OR = 3.68, 95% CI = 1.84–7.36) than in those with normal BMI. With the increase in age, the risk of abnormal BP and PTA of workers showed an upward trend. Compared with male workers, female workers had a lower risk of abnormal PTA (OR = 0.28, 95% CI = 0.16–0.50). Workers with excessive occupational noise exposure had a higher risk of abnormal PTA than their counterparts (OR = 1.86, 95% CI = 1.31–2.62). Model 2 showed that after adjusting for age, sex, and BMI, mining workers had a higher risk of abnormal ECG than manufacturing workers (OR = 1.54, 95% CI = 1.07–2.24). The risk of abnormal PTA increased gradually with the increase in noise exposure value, the risk of abnormal PTA for workers with noise value group ≥90 dB was higher than that for workers with noise value group <80 dB (OR = 4.67, 95% CI = 2.46–8.87) **(Table 2)**.

**Table 2:** Multivariate logistic regression analysis of occupational health examination results.

Dependent variable	Model 1 (n = 1125)				Model 2* (n = 1051)			
	Independent variable	OR	95% CI	*p*-Value	Independent variable	OR	95% CI	*p*-Value
ECG	Noise exposure				Industry category			
	Normal	Reference			Manufacturing	Reference		
	Excessive	0.71	0.53–0.94	0.02	Mining	1.54	1.07–2.24	0.02
					Others	0.35	0.12–1.05	0.06
					Noise value grouping			
					<80	Reference		
					80–84.9	0.24	0.16–0.37	<0.01
					85–89.9	0.46	0.31–0.70	<0.01
					≥90	0.39	0.24–0.62	<0.01
BP	Enterprise size							
	Micro	Reference						
	Small	0.96	0.63–1.47	0.8				
	Medium	0.36	0.19–0.66	**<0.01**				
	Age							
	<40	Reference						
	40–49.9	2.41	1.56–3.73	**<0.01**				
	≥50	3.76	2.47–5.71	**<0.01**				
	BMI (kg/m^2^)							
	≤24.9	Reference						
	25–29.9	1.99	1.49–2.66	**<0.01**				
	≥30	3.68	1.84–7.36	**<0.01**				
PTA	Industry category				Industry category			
	Manufacturing	Reference			Manufacturing	Reference		
	Mining	1.79	1.25–2.55	**<0.01**	Mining	1.41	0.97–2.06	0.08
	Others	1.91	0.75–4.86	0.2	Others	6.31	2.23–17.87	**<0.01**
	Enterprise size				Enterprise size			
	Micro	Reference			Micro	Reference		
	Small	1.65	0.95–2.88	0.08	Small	1.53	0.79–2.96	0.2
	Medium	0.54	0.24–1.21	0.1	Medium	0.56	0.23–1.37	0.2
	Sex				Noise value grouping			
	Male	Reference			<80	Reference		
	Female	0.28	0.16–0.50	**<0.01**	80–84.9	2.88	1.59–5.23	**<0.01**
	Age				85–89.9	3.85	2.13–6.95	**<0.01**
	<40	Reference			≥90	4.67	2.46–8.87	**<0.01**
	40–49.9	4.38	2.40–7.99	**<0.01**				
	≥50	5.11	2.87–9.11	**<0.01**				
	Noise exposure							
	Normal	Reference						
	Excessive	1.86	1.31–2.62	**<0.01**				

* Model 2 adjusted for age, sex and BMI

## 4. Discussion

In this study, we investigated the health status of workers exposed to occupational noise in China. The results of the study found that 53.8% (605/1125) of the workers’ physical examination results contained at least one abnormal condition, and the prevalence rates of abnormal ECG, BP, and PTA were 21.9% (246/1125), 27.8% (313/1125), and 18.0% (202/1125), respectively. The prevalence of abnormal BP and PTA was higher than 17.4% and 8.0% reported in other studies, and ECG was lower than 36.4% in other studies but higher than 13.3% in nonexposed workers in other studies [12]. Factors, such as age, enterprise size, and workplace noise exposure, were strongly associated with worker’s health. The health of workers exposed to occupational noise is not ideal. Relevant agencies and departments must focus on the harm of occupational noise to workers and formulate and adopt corresponding prevention and control measures as soon as possible to reduce the negative effects of noise.

This study found that age was an extremely important influencing factor. With the increase in age, the prevalence of abnormal BP and PTA of workers gradually increased. Similar results were observed in other studies; older workers have lower basal metabolism, organ aging, and dysfunction compared with their younger counterparts [32, 33]. In this work, males were more likely to experience hearing loss than females, which was consistent with the results of other studies [12, 14]. Some studies have found that females are more sensitive to high-frequency sounds compared with males, which may be due to genetic factors. With the increase in age, males experience more rapid auditory decay and more severe hearing loss due to occupational noise exposure than females, which may be because males have more pressure, more poor living habits (such as smoking and drinking), and pay less attention to their hearing compared with females; these factors exacerbate male hearing loss [34]. At age 60, age-related hearing loss (in the 3–6 kHz range) is about 30–40 dB for males and 20 dB for females [14].

Enterprise size was also found to be related to the abnormal BP of workers. Compared with workers in micro enterprises, workers in medium-sized enterprises were less likely to have abnormal BP. This condition may be because micro enterprises ignore the occupational health education for employees, the protective measures have not been implemented, and the safety awareness of employees is relatively weak [35, 36]. Other studies have also shown that employees in micro enterprises have a higher risk of hypertension than those in other enterprises, partly because they are more likely to engage in heavy physical labor and face greater occupational stress [11]. Mining workers have a higher prevalence of abnormal PTA than manufacturing workers possibly because mining workers are more exposed to high levels of occupational noise, such as blasting and rock excavation.

The results showed that excessive noise exposure appeared to be a protective factor for abnormal ECG in workers, contrary to the conclusions of other studies [7, 37]. We speculated that this condition may be due to the lack of sample size and the “healthy worker effect”. Some patients with obvious symptoms were transferred or resigned, so they were not included in the study [38–40]. The noise exposure results were not associated with abnormal BP in workers, but other studies found that exposure to noise ≥80 dB can increase the risk of hypertension in workers by 0.81 times [41].

The results of multivariate logistic analysis showed that noise and hearing loss had a certain dose–response relationship. Noise is an important risk factor for hearing loss in workers. Workers exposed to noise exceeding the occupational exposure limit are more likely to have an abnormal PTA, which suggests that we should focus on the prevention and control of noise. Factors affecting the hearing loss of workers caused by noise are related to the physical fitness of workers and the nature of the noise itself, such as high-frequency noise and low-frequency noise, impulse noise, and steady noise, where the former is more harmful [16]. In addition, NIHL often has no clear symptoms in the early stage, and workers do not pay attention to it. The study found that about a quarter of US adults who self-reported good hearing had audiometric evidence of possible hearing loss [42]. Long-term exposure to noise reduces workers’ attention and awareness, which may lead to safety accidents. Therefore, a sound rest system and hearing-related occupational health examinations are essential in formulating workers’ noise prevention and control strategies.

Each country also has different measures to deal with various levels of occupational noise. Taking China as an example, when the occupational noise in the working environment is 80 dB ≤ *L*_EX,8h_/*L*_EX,W_ <85 dB, health monitoring of workers is required [31]. If 85 dB≤ *L*_EX,8h_/*L*_EX,W_ <90 dB, the degree of hazard is defined as “mild hazard,” so measures need to be taken to reduce workers’ exposure levels, such as setting up noise hazard protection signs, wearing noise protective equipment, and providing occupational health education. If 90 dB≤ *L*_EX,8h_/*L*_EX,W_ <95 dB, the hazard level is defined as “moderate hazard,” and corrective and management actions are necessary based on the characteristics of the enterprise. Moreover, 95 dB ≤ *L*_EX,8h_/*L*_EX,W_ <100 dB and *L*_EX,8h_/*L*_EX,W_ ≥100 dB are designated as “severe hazard” and “extreme hazard,” respectively. In addition to the above measures, corresponding engineering and technical measures must be adopted for rectification. After rectification is completed, the health evaluation and noise classification of the noise control and protection effects will be carried out.

The research findings demonstrated that the prevalence of abnormal BP and ECG were higher than the prevalence of abnormal PTA in workers exposed to occupational noise, indicating that the impact of noise on the cardiovascular system may be more significant than that of the auditory system. As a potential risk factor for cardiovascular disease, noise can cause significant harm by hastening heart aging and elevating the incidence of myocardial infarction, to some degree. However, in contrast to extensive study on its damaging effect on auditory system, noise’s damage on non-auditory system has not attracted widespread attention. Individuals may be more concerned about hearing loss that poses a direct and consequential effect on their health and daily living, but for abnormalities in BP and ECG, it may be perceived as having a less tangible impact on themselves, leading to limited attention. This suggests that we should pay more attention to the cardiovascular disease of workers, find the early risk of disease, and improve the health of workers.

A hygienic and safe working environment, good working habits, regular occupational health examinations, and a sound occupational health monitoring system are the key factors to reduce the effects of noise on workers’ health. To protect the occupational health of workers, we can refer to the “tertiary prevention strategy” to formulate corresponding prevention and control measures. “Primary prevention” should focus on reducing the noise value in the workplace, including the adoption of transformation, replacement of sound sources, and engineering measures, such as the installation of barriers, the use of sound-deadening materials, and zoning measures. Equally important measures include occupational protection for workers, as well as occupational health education and supervision of the use of protective items, such as earplugs and earmuffs. Ensuring that workers have adequate nutritional supplies and sufficient rest and sleep time can reduce the damage of noise on workers’ health. “Secondary prevention” should focus on workers’ occupational health examinations. The government and enterprises should implement an occupational health examination system for workers to facilitate “early detection, early diagnosis, and early treatment” of workers’ health damage and reduce the adverse consequences of hearing loss and cardiovascular disease. “Tertiary prevention” should focus on clinical prevention. For workers who have been clearly diagnosed with cardiovascular disease and hearing loss, including occupational noise-induced deafness, timely and effective treatment should be adopted to prevent the deterioration of the condition, promote functional recovery, and prevent complications and disability. For those workers who have lost the ability to work, rehabilitation therapy measures are required to restore or retain functions as much as possible so that they can participate in social activities and prolong life.

This study had some limitations that should be addressed in follow-up studies. This study had a cross-sectional design. Although it showed an association between the independent variables and the dependent variable, it could not prove the causality between them. The PTA method was adopted to measure the hearing loss in this study. The otoacoustic emission examination is better than PTA in the early identification of hearing loss caused by noise, which can be further improved in future occupational health examinations. Workers may also be exposed to occupational risk factors other than noise, such as ototoxic chemicals and high temperatures, which may have a synergistic effect with noise. This study lacked information about workers’ smoking and drinking habits, which were closely related to workers’ health. There may be a certain correlation between age and exposure time, which needs further exploration in future research. The impact of noise on workers’ auditory and non-auditory systems may not require such high levels of noise exposure, and further research is needed to explore the effects of noise on workers’ health. Another limitation was related to the healthy worker effect because some workers with remarkable hearing loss and cardiovascular disease symptoms may have left their jobs and were not included in our study. In addition, we also provide the research data to provide some reference for other research (**S1 Dataset**).

## 5. Conclusion

The results of this study showed that the prevalence of abnormal ECG, BP, and PTA was high among noise-exposed workers. Factors, such as age, noise level in the workplace, enterprise size, and sex, were related to workers’ health abnormalities. Governments, enterprises, and individuals need to pay great importance to the adverse effects of noise and actively adopt various effective protection measures to ensure the occupational safety and health of workers.

## Supporting information

S1 Fig Flowchart of the enrolled study subjects. (TIF)

S1 Table Table of exposure by characteristics. (XLSX)

S1 Dataset Raw data. (XLS)
