# Supplementary figures and images for: Cross-sectional study on the health of workers exposed to occupational noise in China
Source: PLoS One. 2024 Jun 25;19(6):e0305576. doi: 10.1371/journal.pone.0305576 (PMC11198845; doi:10.1371/journal.pone.0305576)

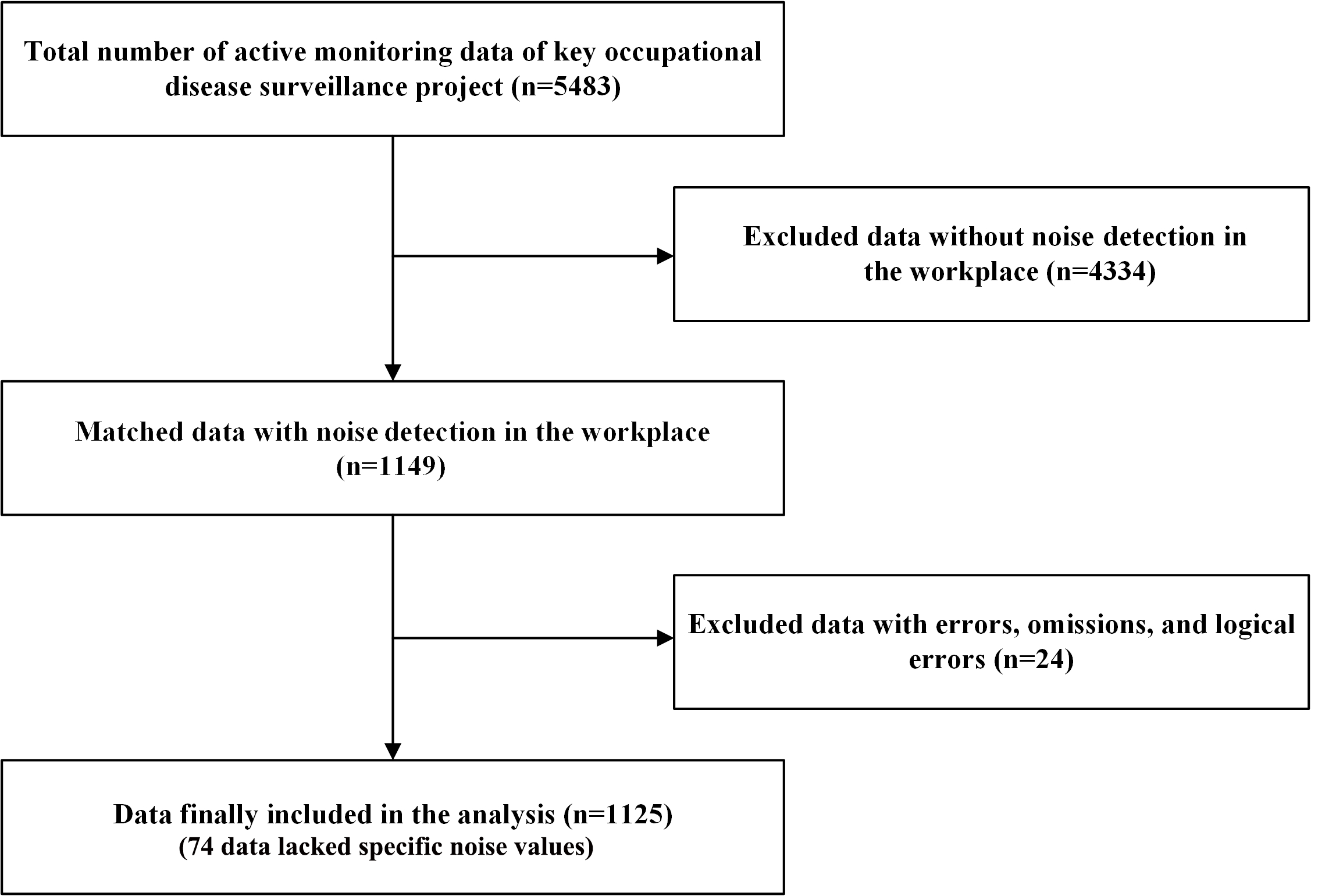

Supplement: S1 Fig — (TIF) [file pone.0305576.s001.tif]
